# Assessing Caregivers’ Skills in Assisting People with Dementia during Mealtime: Portuguese Cultural Adaptation of the Feeding Skills Checklist

**DOI:** 10.3390/ijerph18126467

**Published:** 2021-06-15

**Authors:** Lígia Passos, João Tavares, Daniela Figueiredo

**Affiliations:** 1 Center for Health Technology and Services Research (CINTESIS.UA), Department of Education and Psychology, Campus Universitário de Santiago, University of Aveiro, 3810-193 Aveiro, Portugal; ligiamaria@ua.pt; 2 Institute of Biomedical Sciences Abel Salazar, University of Porto, 4099-002 Porto, Portugal; 3 Center for Health Technology and Services Research (CINTESIS.UA), School of Health Sciences, Campus Universitário de Santiago, University of Aveiro, 3810-193 Aveiro, Portugal; joaoptavares@ua.pt

**Keywords:** dementia, mealtime difficulties, nursing assistants, caregiver, assessment tool, reliability, content validity

## Abstract

In advanced dementia, individuals usually develop feeding difficulties. The Feeding Skills Checklist (FSC) is an instrument to assess caregivers’ skills when assisting people with dementia (PwD) at mealtimes. This study aimed to adapt and culturally validate a European Portuguese version of the FSC (FSC-PT) and test its reliability. Initially, translation and cultural validation of the FSC, with estimation of the content validity index (CVI), was conducted. Then, the FSC was applied to 23 female nursing assistants (mean age 44.73 ± 10.42 years) while offering lunch (n = 41 meals) to institutionalized PwD. Inter-rater reliability was determining using Cohen’s Kappa. FSC-PT showed excellent content validity, with item-content validity index ranging from 0.85 to 1, scale level average CVI = 0.94 and universal agreement CVI = 0.54. It also showed very satisfactory inter-observer reliability, with Cohen’s Kappa = 0.844. Of the 41 meals analyzed, only 37.7% of the actions/good practices in feeding PwD were observed. A positive and moderate correlation was found between the length of time working as nursing assistance and the FSC environment dimension (r_s_ = 0.435; *p* = 0.038). The results supported the content validity of the FSC-PT, which shows considerable potential to be an instrument for verifying caregivers’ skills when helping PWD to eat and should be increasingly studied.

## 1. Introduction 

In a world with a growing and accelerating increase in the number of cases of dementia, it is common to observe that, in advanced stages, people manifest feeding difficulties characterized by swallowing disorders, refusal to eat, food retention in the mouth or even a negative interaction with the caregiver during mealtime [1,2,3]. Eating tends to be one of the last activities of daily living (ADLs) to be lost by older people [4]. In cases of dementia, because of the damaged cognitive function, this occurs at the more advanced stages, affecting the performance of self-feeding and leading to the need to be fed by a provider [5]. Maintaining nutritional status becomes a challenge to family members and health professionals, who often need to decide on an alternative diet/hydration route [6]. However, in advanced dementia, international scientific societies do not recommend tube feeding [7,8,9,10,11] and recent studies do not prove its benefits, suggesting a preference for an adapted and careful oral handfeeding [12].

In Portugal, it is estimated that 182,000 people live with dementia [13], with about 19.9 cases per thousand inhabitants, being the 4th country with more cases in the world [14]. In 2018, about 98,331 older people lived in Portuguese nursing homes [15], but it is unknown how many of these have some type of dementia. This number can be high, since it is estimated that, in industrialized countries, 60% of institutionalized people have some form of dementia [16]. 

The caring of institutionalized people with dementia (PwD) is delivered by employees with low education and little specialized training, in addition to low knowledge about health and rehabilitation needs of this population [17]. Nursing assistants with little skills and no specific training are not able to correctly identify the feeding difficulties and needs of PwD, as well as to provide adequate assistance [18]. When PwD with feeding difficulties do not receive the necessary support, it can mainly result in malnutrition, dehydration, weight loss, and/or feeding tube placement [19,20]. A skilled caregiver is expected to have a positive attitude, patience, and good interaction with PwD, in addition to an increased sensitivity to control perceived inappropriate behaviors [18], thus leading to an increase in PwD’s food intake at mealtime, which also becomes a most pleasant moment for the PwD and caregiver dyad [21]. By evaluating caregivers’ skills when providing assistance during mealtimes, it is possible to realize their weaknesses and strengths that interfere with PwD’s feeding performance. Recognizing weaknesses allows designing training programs to improve knowledge and self-efficacy to provide better mealtime assistance [22].

Usually, institutional care takes place in environments where the focus is only on performing a task (offering lunch, for example), minimizing many other components that caring involves [23]. A social-ecological model (SEM) inserts individuals into broad social systems and describes their interactive characteristics with the underlying environments as influencing health outcomes [24]. A SEM applied to feeding difficulties can assist in the development of systematic intervention mechanisms capable of changing behavior in several dimensions (intrapersonal, interpersonal, environmental and institutional), optimizing the health and well-being of PwD [25,26]. Framing the mealtime of people with feeding difficulties from a social-ecological perspective allows better identification of the points of personal and environmental change for effective and sustainable interventions to improve the food intake [25].

The Feeding Skills Checklist (FSC) consists of a checklist, developed by Melissa Batchelor [22], one of the creators of a model called C3P, an acronym for “change the person, change the people and/or change the place”. Based on a SEM, this model aims to adjust care strategies to promote independence and, at the same time, provide adequate support as PwD present cognitive and functional deterioration [23]. Supported by the three main perspectives of a SEM, the C3P model considers that eating goes beyond being a simple activity of daily living, so when a PwD has feeding difficulties, the caregiver must look beyond food, and also to factors related to their interaction with the person and also to the environmental conditions that are favorable to a safe and pleasant meal [27].

The FSC was developed based on literature review, clinical experience and current clinical practice guidelines [22]. It consists of 24 items that assess the skills of long-term care institutions’ staff when assisting residents with dementia at mealtimes. The items, which are considered good feeding practices, are categorized in three dimensions, according to the C3P model: Person (with dementia)—10 items that assess patients’ food preferences and meal routines, as well as their conditions to receive a meal; People (caregiver): 7 items that assess the skills of the caregiver and his/her interaction with PwD during a meal, and Place (environment): 7 items that assess the conditions of the dining room that favor the PwD to eat better. During the period of the meal to be observed, the presence or absence of the content specified in each item must be checked, or even if the item does not apply to the situation to be observed [22]. Although there is no established way to account for the final score of the application of the FSC, these authors consider the attribution of one point to each action performed in the checklist.

To date, there has been no published study on the reliability or validity of the FSC, nor even a translation into other languages. The only work published to date, which made use of the FSC, aimed to assess the feasibility of a virtual training for caregivers on skills to feed people with dementia [22]. The FSC was applied to 35 employees of nursing homes in the United States, however, no assessment of the checklist properties has been made, and the study’s results only demonstrate the improvement in the average of the good actions performed after the staff training.

The present study aimed to adapt and culturally validate a European Portuguese version of the Feeding Skills Checklist and test its reliability. The feeding practices performed by the participants were briefly characterized, as well as an analysis of the associations between the sociodemographic variables and the FSC results.

## 2. Materials and Methods

### 2.1. Design and Participants

This study was designed as an observational, transversal, exploratory-descriptive study, with a convenience sample of nursing assistants (NA) selected from nursing homes (NH) in the central region of Portugal. It started with a process of translation, cultural adaptation, and linguistic validation. Then, an analysis of its interobserver reliability was conducted. This study included (1) nursing assistants directly involved in the assistance with meals, (2) hired for at least three months at the nursing home, and (3) who accepted to participate voluntarily by signing the consent form. Nursing assistants who were in a professional internship situation were excluded. Data were collected between September and November 2019. The Ethics Committee of the Health Sciences Research Unit—UICISA: E approved this study (Ref. 598/06-2019).

### 2.2. Translation and Adaptation Procedures

The process of development of the European Portuguese version of the Feeding Skills Checklist (FSC-PT) started with the authorization of its author. The principles of good practice for translation and cultural adaptation proposed by the International Society for Pharmacoeconomics and Outcomes Research (ISPOR) were adopted [28].

The first author translated the Feeding Skills Checklist (FSC) into the Portuguese language, and then, the co-authors made a reconciliation. The first version of the FSC-PT was achieved after review and adaptation of the initial translation, and discussion between the authors for consensus. A faculty professor, an English native speaker with fluency in European Portuguese, performed a retroversion into English. In the harmonization stage, the versions were compared in an item-by-item analysis made by all translators, resulting in a second version of the FSC-PT. To check the comprehensibility, interpretation and cultural relevance of the translation [28], and to verify the understandability and meaning of the translated items, a convenience sample consisting of one nurse, three gerontologists, one psychologist, one social worker and two speech therapists were invited to join in the cognitive debriefing stage (n = 8). The expert panel was chosen according to academic degree, knowledge, and experience on the subject of the study. They were advised to evaluate the characteristics of semantic, idiomatic, and experiential or cultural equality, and then to indicate the degree of agreement between the FSC original version and the Portuguese translated version on a scale ranging from 1 to 4, which corresponds to 1 = disagree, 2 = slightly agree, 3 = agree and 4 = totally agree. If 1 or 2 were chosen, the experts had to explain their choice and to leave comments and suggestions in a specific area. To consider consensus among the experts, an agreement of ≥80% was required. The items with <80% of agreement were re-evaluated in a second round and the experts were able to maintain or change their opinion. By the end of this stage, version 3 of the FSC-PT was established. To conclude, an orthographic revision was done by a Portuguese language teacher and the final version of the FSC-PT was created.

The level of satisfaction of the panel of experts when assessing an instrument was measured through the content validity index (CVI) according to Polit and Beck [29]. The CVI was computed calculating each item CVI (I-CVI), which is determined by combining the items ranked as 3 or 4 to be divided by the total number of responses and by scale-level-CVI (S-CVI), which is calculated by two methods: Universal Agreement among experts (S-CVI/UA) and the Average CVI (S-CVI/Ave). The S-CVI/UA is measured by the number of items with I-CVI equal to 1 divided by the total number of items, and the S-CVI/Ave by taking the sum of all I-CVIs divided by the total number of items [30]. The probability of change (PC) and Kappa (κ) were also determined, which is a consent index of the agreement amongst the evaluators that complements the CVI, removing the random chance agreement [31].

### 2.3. Assessment of the Psychometric Properties of the FSC-PT

This part of the study was conducted in six nursing homes in Central Portugal and involved the application of the FSC-PT. The behavior of 23 nursing assistants was observed while they offered lunch to people with dementia (PwD). A total of 41 meals were observed (sometimes the same NA offered lunch to more than one person). Thirty meals were monitored at the same time by the leading researcher as well as a psychologist with academic training in gerontology and geriatrics and trained to use the checklist, to posteriorly examine the interobserver reliability.

### 2.4. Statistical Analysis

The sample was characterized using descriptive statistics through the estimate of absolute frequencies and measures of central tendency and dispersion (mean and standard deviation). The interobserver reliability was measured by Cohen’s Kappa, whose values range between 0 and 1, being <0 = poor, 0.00–0.20 = weak, 0.21–0.40 = considerable, 0.41–0.60 = moderate, 0.61–0.80 = substantial and 0.81–1.00 = almost perfect [32]. The percentage of agreement between the observers was also determined.

For the study of the relationship between sociodemographic variables and the total FSC score, the normality assessed by the Shapiro–Wilk test was not verified, and nonparametric tests were adopted: Mann–Whitney U test for comparison of difference group means’ independent variables and Spearman’s coefficient to verify the correlation between two variables. Values of *p* < 0.05 were considered significant. Data analysis was performed using the statistical software SPSS 25.0 version (IBM Corp., Armonk, NY, USA).

## 3. Results

### 3.1. Content Validity

Upon conclusion of the first evaluation round, 12 items had an I-CVI < 0.8. After the second round, all items showed an increase in I-CVI, being higher than 0.8. The mean value of the I-CVI in the conclusion of the second round of evaluations was 0.945. The S-CVI/UA obtained was 0.54 and the S-CVI/AVE was 0.94. The PC values ranged between 0.004 and 0.218. The modified Kappa varied between 0.52 and 1, with an average κ = 0.93 (Table 1).

### 3.2. Participants

The sample consisted of 23 nursing assistants, all females. The mean age was 44.73 years (SD = 10.42). The participants have worked as NA for 10.28 years (SD = 10.46) in the profession, most of them had low academic qualifications and 56.5% reported having received specific training on the feeding difficulties of people with dementia (Table 2).

### 3.3. Interobserver Reliability

To determine the FSC-PT interobserver reliability, a sample of 30 observed meals was analyzed. The value of Cohen’s Kappa was 0.844 and the percentage of agreement between observers was 94.14% (Table 3).

### 3.4. Study of the Good Practices in Feeding

The interaction of the caregiver-PwD dyad was observed in 41 meals. A mean of 37.7% of the actions, which are considered good practices in feeding PwD, were observed. The “person with dementia” dimension obtained the lowest percentage of actions performed, with only 21.2%. The dimensions “caregiver” and “environment” had similar results, with 50.4% and 49% respectively.

**Figure 1:**
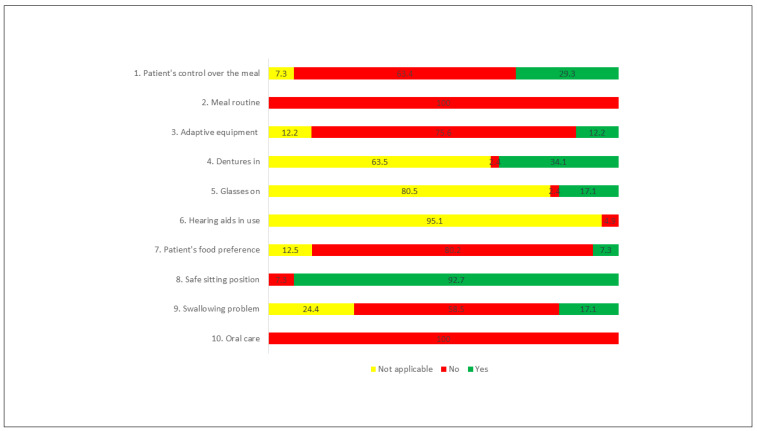
Actions performed (%)—Person with Dementia dimension.

Among the items of the FSC-PT, in the “person with dementia” dimension, the most performed action was to maintain the person in a seated and safe position, performed in 92.7% of the observed meals. In contrast, the existence of a meal routine (e.g., washing hands, praying before eating) and the performance of oral hygiene after the meal were not performed in any of the 41 meals observed. In 80.2% of the meals, the resident was not asked what she/he preferred to eat first (Figure 1).

**Figure 2:**
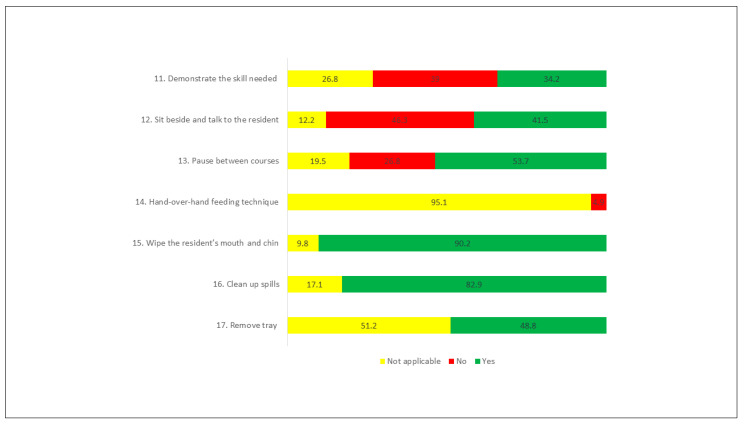
Actions performed (%)—Caregiver dimension.

In the “caregiver” dimension, the most performed actions were items 15 (Wipe the resident’s mouth and chin, as needed during the meal), and 16 (Clean up any spills and change linen, as necessary) being performed, respectively, in 90.2% and 82.9% of the meals observed. The least performed action was item 11 (Demonstrate the skill needed), performed only 34.2% of the time (Figure 2).

**Figure 3:**
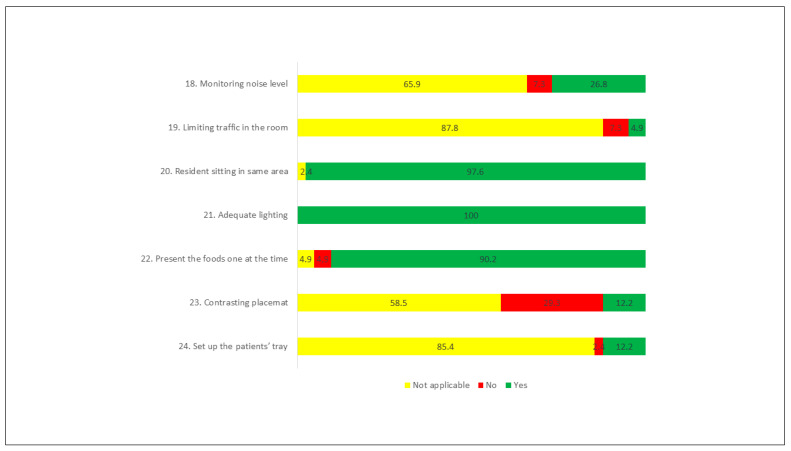
Actions performed (%)—Environment dimension.

In the “environment” dimension, there was a greater number of actions taken. In all the meals observed, the lighting in the dining room (item 21) was adequate. In 97.6% of the time, it was registered that the PwD sat in the same area for each meal, and the food was presented one at time in 90.2% of the observed meals. The action to limit movement around the dining room occurred in only 4.9% of the observations. It was in this dimension that the “not applicable” answer option was attributed the greatest number of times (Figure 3).

The variables of age, length of time working as NA, workload, education, and specific training on the feeding difficulties of PwD did not show any statistically significant associations or differences with the percentage of actions performed of the FSC-PT. However, the length of time as NA worker and the FSC-PT environment dimension showed a positive and moderate correlation (r_s_ = 0.435; *p* = 0.038), i.e., those with more years of profession performed a greater amount of good practices regarding the environmental dimension.

## 4. Discussion

The purpose of this study was to translate and adapt the Feeding Skills Checklist into the Portuguese language, and to evaluate its interobserver reliability, in addition to characterizing feeding practices by nursing assistants when caring of institutionalized PwD, comprising an analysis of the correlation of the participants’ sociodemographic variables and the results of FSC-PT. After a thorough process of translation, adaptation and linguistic validation, the analysis of CVI included an individual analysis of each item (I-CVI) and a global checklist (S-CVI/UA and S-CVI/AVE). These approaches were chosen to exclude the effect of outliers in the analysis of global values. The translation and linguistic validation process of assessment instruments requires a robust methodology and the CVI assessment is a key part of ensuring the quality of studies. Several studies on the creation and adaptation of assessment scales and instruments have resorted to the calculation of the CVI [33,34,35].

The Portuguese version of the FSC presented a mean I-CVI of 0.937, which is considered appropriate when an instrument is evaluated by an expert panel composed of more than 6 participants (CVI > 0.8) [29,36], as well as in the evaluation of new instruments (CVI > 0.9) [29]. The S-CVI/UA obtained was 0.54, and the S-CVI/AVE was 0.94. Overall, the Universal Agreement showed moderate content validity while the Average approach shows excellent content validity of the FSC [15]. When determining the CVI, it is known the degree of relevance and representativeness that each item has on a construct and its specific objective of evaluation, therefore, the study of content validity is needed for the development or adaptation of evaluation instruments [37,38,39,40]. As a quantitative measure of content validity, CVI evaluates the proportion of experts who agree on the instrument’s items and the linguistic validation process ends when it reaches a value equal to or greater than 0.8 for all items [41]. It is absolutely important that the expert panel evaluation be part of the cultural adaptation process of an instrument. Some authors suggest a number of 5 to 10 members [36] and others recommend at least 6 and a maximum of 20 experts [37]. In this study, the academic degree, qualification, and availability of the members of the expert panel were considered, given the characteristics and thematic of the FSC. The experts were chosen based on their clinical and/or research experiences, privileging multidisciplinarity, as suggested by Alexandre and Coluci [41]. All eight experts who contributed to this study had PhDs in social sciences or health sciences, and experience in the fields of aging and/or dementia, thus allowing the instrument to have a global, prudent, and reliable assessment. The criteria for choosing the experts were essential, as dementia is a topic that requires varied and specific knowledge, including the presence of professionals who work directly with feeding issues in aging, such as nurses and speech therapists.

This study continued with an assessment of the psychometric property of the FSC-PT, which shows the quality and scientific value of the results found after using an assessment instrument [42]. As these instruments are important for researching and also for clinical care practice, assessing their qualities ensures the availability of a consistent tool for both researchers and healthcare professionals [42].

The FSC-PT interobserver reliability is satisfactory, with Cohen’s Kappa = 0.844 and 94.14% agreement. The lack of previous studies that assess psychometric properties of the FSC makes comparisons unfeasible. It is noteworthy that some items had much lower Kappa values, such as items 4, 5 and 6 of the PwD dimension, which concern the placement and use of dentures (Kappa = 0.670), glasses (Kappa = 0.551) and hearing aids (Kappa = 0.193) and item 14, of the caregiver dimension, which refers to the use of a specific feeding technique hand-over-hand (Kappa = 0.651). These items generated doubts between the options “no” and “not applicable”. As the limitations of the senses represent one of the main causes of disability in older people [43], despite the low Kappa values, it was decided to keep these items on the checklist. However, future studies may create an FSC application manual so that the evaluator has clear references on what to observe, to prevent bias in the interobservation. The use of handfeeding techniques, such as “hand-over-hand” has been studied by the creator of the FSC [2,22,44] along with how the results point to the improvement of food intake of the older PwD, therefore, it was also decided to keep the item. In studies involving an intervention program, this technique could be taught to caregivers. It is suggested to create an FSC application manual, to make it clear that if this technique is not part of the institution’s procedures, the option “not applicable” should be checked.

After exploring the FSC interobserver reliability, this study was complemented with the use of the checklist to a sample of nursing assistants, in order to describe the feeding practices performed in NH where these participants work. The sample formed by 23 NA performed an average of only 37.7% of FSC’s actions during the observed meals. The dimensions “caregiver” and “environment” had similar and better results than the dimension “person with dementia”, with 50.4% of the actions focusing on the caregiver, 49% of the actions related to the environment and only 21.2% of actions directed to the PwD. These data suggest a weakness in the interpersonal relationship between NA and PwD, as well as a greater control of environmental and institutional issues.

Only the profession time variable showed a statistically significant correlation with the FSC environment dimension (r_s_ = 0.435, *p* = 0.038), which may indicate that professional experience makes the NA better able to control environmental factors, making the dining room more appropriate for older PwD to eat better. The variables age, length of time working as NA, workload, education, and specific training on the feeding difficulties of PwD did not show any statistically significant associations or differences with the percentage of actions performed at FSC-PT. The variables analyzed in this study were insufficient to characterize the sample well and to assess the existence of other significant correlations, since the data of the participants were mostly relative to the intrapersonal aspects, thus limiting a complete analysis of the dimensions of a SEM. Future studies should include specific assessment instruments (e.g., to assess NA workload) or explore other variables that may influence the actions of the three dimensions of FSC, as they have presented such low results.

A limitation of this study is the small convenience sample size, which limits the generalization of the results to the Portuguese population of NA in NH. Future studies should be conducted with larger samples and the participants can be better characterized, considering variables based on the social-ecological model, in addition to using a regression model to analyze the most significant variables. A better characterization of the institutions could also contribute to analyze the impact of institutional policies on the results of the FSC-PT, especially in the environmental dimension.

Given the excellent content validity of the FSC-PT evidenced by the present study, future research should assess the construct validity (e.g., confirmatory factor analysis) of the three dimensions of the checklist (person with dementia, caregiver, and environment). Concurrent and discriminant validity, as well as psychometric properties, can also be analyzed so that the effectiveness of the FSC-PT in recognizing the strengths of the caregivers and also the weaknesses that occur when helping at the PwD mealtime can be known.

## 5. Conclusions

The foremost objective of this study was to contribute to the adaptation and validation of the FSC for the Portuguese language, to study its interobserver reliability, and to correlate the sociodemographic variables with the results of the checklist. Through a rigorously linguistic validation procedure and a reliability measure, the results indicate that the FSC-PT allows to identify (and characterize) the performance of good practices in helping PwD with meals.

The FSC-PT shows great potential to be an instrument for verifying good practices carried out when helping a person with dementia to eat and should be increasingly studied. The findings showed that higher CVI, PC and Kappa of FSC supported the content validity of the Portuguese version of FSC. More than an assessment tool, it allows the staff to adapt procedures and actions to cover inter/intrapersonal, environmental, and institutional aspects involved in mealtime. Future studies should evaluate the psychometric properties and other types of validity of this scale.

## Figures and Tables

**Table 1:** Analysis of FSC-PT content validity.

FSC Dimension	Item	I-CVI		PC		κ		Interpretation	
		1st Round	2nd Round	1st Round	2nd Round	1st Round	2nd Round	1st Round	2nd Round
**Person with dementia**	1. Patient’s control over the meal	0.625	1	0.218	0.004	0.52	1	Fair	Excellent
	2. Meal routine	0.625	1	0.218	0.004	0.52	1	Fair	Excellent
	3. Adaptive equipment	0.625	0.85	0.218	0.12	0.52	0.72	Fair	Good
	4. Dentures in	1	-	0.004	-	1	-	Excellent	Excellent
	5. Glasses in	1	-	0.004	-	1	-	Excellent	Excellent
	6. Hearing aids in use	0.75	1	0.12	0.004	0.72	1	Good	Excellent
	7. Patient’s food preference	0.625	1	0.218	0.004	0.52	1	Fair	Excellent
	8. Safe sitting position	0.625	1	0.218	0.004	0.52	1	Fair	Excellent
	9. Swallowing problem	0.625	0.875	0.218	0.0313	0.52	0.87	Fair	Excellent
	10. Oral care	0.875	-	0.0313	-	0.87	-	Excellent	Excellent
**Caregiver**	11. Demonstrate the skill needed	0.75	0.875	0.12	0.0313	0.72	0.87	Good	Excellent
	12. Sit beside and talk to the resident	0.875	-	0.0313	-	0.87	-	Excellent	Excellent
	13. Pause between courses	0.75	0.875	0.12	0.0313	0.72	0.87	Good	Excellent
	14. Hand-over-hand feeding technique	0.875	-	0.0313	-	0.87	-	Excellent	Excellent
	15. Wipe the resident’s mouth and chin	0.75	0.875	0.12	0.0313	0.72	0.87	Good	Excellent
	16. Clean up spills	0.625	1	0.218	0.004	0.52	1	Fair	Excellent
	17. Remove tray	0.75	1	0.12	0.004	0.72	1	Good	Excellent
**Environment**	18. Monitoring noise level	1	-	0.004	-	1	-	Excellent	Excellent
	19. Limiting traffic in the room	1	-	0.004	-	1	-	Excellent	Excellent
	20. Resident sitting in same area	0.875	-	0.0313	-	0.87	-	Excellent	Excellent
	21. Adequate lighting	1	-	0.004	-	1	-	Excellent	Excellent
	22. Present foods one at a time	0.875	-	0.0313	-	0.87	-	Excellent	Excellent
	23. Contrasting placemat	1	-	0.004	-	1	-	Excellent	Excellent
	24. Set up the patient’s tray	0.875	-	0.0313	-	0.87	-	Excellent	Excellent
	**Mean**	0.807	0.945						

PC was calculated using the formula [N!/A! (N − A)!] where N = the number of experts and A = the number of experts who agree that the item is relevant. Kappa was calculated using the formula κ = CVI − PC/(1 − PC). Interpretation criteria for κ [29,31]: fair = κ from 0.40 to 0.59; good = κ from 0.60 to 0.74; excellent = κ > 0.74. CVI, content validity index; FSC-PT, Portuguese version of the Feeding Skills Checklist; PC, probability of change.

**Table 2:** Sociodemographic data (n = 23).

Variable	n = 23
**Age** [years] Mean (SD)	44.73 (10.42)
**Profession time** [years] Mean (SD)	10.28 (10.46)
**Time in the NH** [years] Mean (SD)	7.81 (8.96)
**Sex** n (%)	
Female	23 (100)
**Education** n (%)	
Primary school	2 (8.8)
Secondary school	14 (60.8)
High school	4 (17.4)
University	3 (13.0)
**Marital Status** n (%)	
Single	2 (8.7)
Married	19 (82.6)
Divorced	2 (8.7)
**Weekly workload** [hours] Mean (SD)	38.17 (1.46)
**Weekly workload** n (%)	
37 h	14 (60.9)
40 h	9 (39.1)
**Specific training** n (%)	
Yes	13 (56.5)
No	10 (43.5)

SD: standard deviation; NH: nursing home.

**Table 3:** FSC-PT interobserver reliability (n = 30).

		Observer 1 n (%)			Observer 2 n (%)			K	*p* Value	C
FSC Dimension	Item	Yes	No	Not Applicable	Yes	No	Not Applicable			
**Person with dementia**	1. Patient’s control over the meal	11 (36.7)	16 (53.3)	3 (10)	11 (36.7)	16 (53.3)	3 (10)	1	<0.01	100
	2. Meal routine	-	30 (100)	-	-	30 (100)	-	*	*	100
	3. Adaptive equipment	4 (13.3)	22 (73.3)	4 (13.3)	4 (13.3)	20 (66.7)	6 (20)	0.857	<0.01	93.3
	4. Dentures in	10 (33.3)	1 (3.3)	19 (63.3)	10 (33.3)	7 (23.3)	13 (43.3)	0.670	<0.01	80
	5. Glasses in	7 (23.3)	1 (3.3)	22 (73.3)	7 (23.3)	9 (30)	14 (46.7)	0.551	<0.01	73.3
	6. Hearing aids in use	-	2 (6.7)	28 (93.3)	-	12 (40)	18 (6)	0.193	0.73	66.6
	7. Patient’s food preference	3 (10)	23 (76.7)	4 (13.3)	2 (6.7)	24 (80)	4 (13.3)	0.724	<0.01	90
	8. Safe sitting position	27 (90)	3 (10)	-	28 (93.3)	2 (6.7)	-	0.783	<0.01	96.6
	9. Swallowing problem	4 (13.3)	19 (63.3)	7 (23.3)	5 (16.7)	16 (53.3)	9 (30)	0.825	<0.01	90
	10. Oral care	-	30 (100)	-	-	30 (100)	-	*	*	100
**Caregiver**	11. Demonstrate the skill needed	7 (23.3)	13 (43.3)	10 (33.3)	7 (23.3)	15 (50)	8 (26.7)	0.896	<0.01	93.3
	12. Sit beside and talk to the resident	9 (30)	17 (56.7)	4 (13.3)	9 (30)	19 (63.3)	2 (6.7)	0.877	<0.01	93.3
	13. Pause between courses	15 (50)	11 (36.7)	4 (13.3)	14 (46.7)	12 (40)	4 (13.3)	0.945	<0.01	96.6
	14. Hand-over-hand feeding technique	-	2 (6.7)	28 (93.3)	-	1 (3.3)	29 (96.7)	0.651	<0.01	96.6
	15. Wipe the resident’s mouth and chin	26 (86.7)	-	4 (13.3)	26 (86.7)	-	4 (13.3)	1	<0.01	100
	16. Clean up spills	24 (80)	-	6 (20)	25 (83.3)	-	5 (16.7)	0.889	<0.01	96.6
	17. Remove tray	17 (56.7)	-	13 (43.3)	15 (50)	-	15 (50)	0.867	<0.01	93.3
**Environment**	18. Monitoring noise level	11 (36.7)	1 (3.3)	18 (60)	11 (36.7)	1 (3.3)	18 (60)	1	<0.01	100
	19. Limiting traffic in the room	2 (6.7)	1 (3.3)	27 (90)	2 (6.7)	1 (3.3)	27 (90)	1	<0.01	100
	20. Resident sitting in same area	29 (96.7)	-	1 (3.3)	29 (96.7)	-	1 (3.3)	1	<0.01	100
	21. Adequate lighting	30 (100)	-	-	30 (100)	-	-	*	*	100
	22. Present foods one at a time	27 (90)	2 (6.7)	1 (3.3)	27 (90)	2 (6.7)	1 (3.3)	1	<0.01	100
	23. Contrasting placemat	1 (3.3)	12 (40)	17 (56.7)	1 (3.3)	12 (40)	17 (56.7)	1	<0.01	100
	24. Set up the patients’ tray	4 (13.3)	1 (3.3)	25 (83.3)	4 (13.3)	1 (3.3)	25 (83.3)	1	<0.01	100
	**Mean**							0.844		94.14

FSC-PT = Portuguese version of the Feeding Skills Checklist; C = percentage of agreement. * No statistical test was calculated because the variable was constant.

## References

[1] Cintra M.T.G., de Rezende N.A., de Torres H.O.G. (2013). Qual a via mais adequada para a alimentação de idosos com demência avançada: Oral ou enteral?. Geriatr. Gerontol. Aging.

[2] Batchelor-Murphy M., Crowgey S. (2016). Mealtime Difficulties in Dementia. Evidence-Based Geriatric Nursing Protocols for Best Practice.

[3] Stone L. (2014). Eating/Feeding Issues in Demenita: Improving the Dining Experience. End Life J..

[4] Morris J.N., Fries B.E., Morris S.A. (1999). Scaling ADLs Within the MDS. J. Gerontol. Ser. A.

[5] Palese A., Grassetti L., Bandera D., Zuttion R., Ferrario B., Ponta S., Hayter M., Watson R. (2018). High feeding dependence prevalence in residents living in Italian nursing homes requires new policies: Findings from a regionally based cross-sectional study. Health Policy N. Y..

[6] Martins A.S., de Rezende N.A., da Torres H.O.G. (2012). Occurrence of complications and survival rates in elderly with neurological disorders undergoing enteral nutrition therapy. Rev. Assoc. Med. Bras..

[7] American Geriatrics Society Ethics Committee and Clinical Practice and Models of Care Committee (2014). American Geriatrics Society Ethics Committee American Geriatrics Society Feeding Tubes in Advanced Dementia Position Statement. J. Am. Geriatr. Soc..

[8] Barrocas A., Geppert C., Durfee S.M., Maillet S.R., Monturo C., Mueller C., Stratton K., Valentine C., Aspen T. (2010). ASPEN Ethics Position Paper. Nutr. Clin. Pract..

[9] Volkert D., Chourdakis M., Faxen-Irving G., Frühwald T., Landi F., Suominen M.H., Vandewoude M., Wirth R., Schneider S.M. (2015). ESPEN guidelines on nutrition in dementia. Clin. Nutr..

[10] Royal College of Physicians (2010). Oral Feeding Difficulties and Dilemmas. A Guide to Practical Care, Particularly Towards the End of Life.

[11] Gonçalves J.T.M., Horie L.M., Elisa S., Batista A., Bacchi M.K., Bailer M.C., Barbosa-silva T.G., Paula A., Barrére N., Barreto P.A. (2019). Diretriz BRASPEN de Terapia Nutricional no Envelhecimento. Braspen J..

[12] Goldberg L.S., Altman K.W. (2014). The role of gastrostomy tube placement in advanced dementia with dysphagia: A critical review. Clin. Interv. Aging.

[13] Alzheimer Europe: The Prevalence of Dementia in Europe. https://www.alzheimer-europe.org/Policy-in-Practice2/Country-comparisons/2013-The-prevalence-of-dementia-in-Europe.

[14] OECD (2018). Care Needed: Improving the Lives of People with Dementia.

[15] Rodrigues I.B., Adachi J.D., Beattie K.A., MacDermid J.C. (2017). Development and validation of a new tool to measure the facilitators, barriers and preferences to exercise in people with osteoporosis. BMC Musculoskelet. Disord..

[16] Kuske B., Luck T., Hanns S., Matschinger H., Angermeyer M.C., Behrens J., Riedel-Heller S.G. (2009). Training in dementia care: A cluster-randomized controlled trial of a training program for nursing home staff in Germany. Int. Psychogeriatr..

[17] Barbosa A.L., Cruz J., Figueiredo D., Marques A., Sousa L. (2011). Cuidar de idosos com demência em instituições: Competências, dificuldades e necessidades percepcionadas pelos cuidadores formais. Psicol. Saúde Doenças.

[18] Chang C.-C., Wykle M.L., Madigan E.A. (2006). The Effect of A Feeding Skills Training Program for Nursing Assistants Who Feed Dementia Patients in Taiwanese Nursing Homes. Geriatr. Nurs. Minneap..

[19] Kuo S., Rhodes R.L., Mitchell S.L., Mor V., Teno J.M. (2009). Natural History of Feeding-Tube Use in Nursing Home Residents With Advanced Dementia. J. Am. Med. Dir. Assoc..

[20] Amella E.J. (2002). Resistance at mealtimes for persons with dementia. J. Nutr. Health Aging.

[21] Simmons S.F., Osterweil D., Schnelle J.F. (2001). Improving food intake in nursing home residents with feeding assistance: A staffing analysis. J. Gerontol. A Biol. Sci. Med. Sci..

[22] Batchelor-Murphy M., Amella E.J., Zapka J., Mueller M., Beck C. (2015). Feasibility of a web-based dementia feeding skills training program for nursing home staff. Geriatr. Nurs. Minneap..

[23] Amella E.J., Batchelor-Aselage M.B. (2014). Facilitating ADLs by caregivers of persons with dementia: The C3P model. Occup. Ther. Health Care.

[24] Golden S.D., Earp J.A.L. (2012). Social Ecological Approaches to Individuals and Their Contexts: Twenty Years of Health Education & Behavior Health Promotion Interventions. Health Educ. Behav..

[25] Shune S.E., Linville D. (2019). Understanding the dining experience of individuals with dysphagia living in care facilities: A grounded theory analysis. Int. J. Nurs. Stud..

[26] Liu W., Williams K., Batchelor-Murphy M., Perkhounkova Y., Hein M. (2019). Eating performance in relation to intake of solid and liquid food in nursing home residents with dementia: A secondary behavioral analysis of mealtime videos. Int. J. Nurs. Stud..

[27] Amella Krug E.J., Qanungo S., Martin K.L., Mueller M., Madisetti M., Kelechi T.J. (2020). A cluster randomized controlled trial to assess the efficacy of a telehealth-based train-the-trainer mealtime intervention delivered by respite care center volunteers to caregivers of persons with dementia to improve nutritional outcomes and quality of li. BMC Nutr..

[28] Wild D., Alyson G., Mona M., Sonya E., Sandra M., Verjee-Lorenz A., Erikson P. (2005). Principles of Good Practice for the Translation and Cultural Adaptation Process for Patient-Reported Outcomes (PRO) Measures. Value Health.

[29] Polit D.F., Beck C.T. (2006). The content validity index: Are you sure you know what’s being reported? Critique and recommendations. Res. Nurs. Health.

[30] Zamanzadeh V., Ghahramanian A., Rassouli M., Abbaszadeh A., Alavi-Majd H., Nikanfar A.-R. (2015). Design and Implementation Content Validity Study: Development of an instrument for measuring Patient-Centered Communication. J. Caring Sci..

[31] Zamanzadeh V., Rassouli M., Abbaszadeh A., Majd H.A., Nikanfar A., Ghahramanian A. (2014). Details of content validity and objectifying it in instrument development. Nurs. Pract. Today.

[32] Landis J.R., Koch G.G. (1977). The Measurement of Observer Agreement for Categorical Data. Biometrics.

[33] Yang Y., Yang Y., Hsiao C., Kuo H., Wang J. (2020). Development and psychometric testing of a dementia care competence scale for nurses working in acute care setting. Scand. J. Caring Sci..

[34] Suvanich R., Chatchawan U., Jariengprasert C., Yimtae K., Hunsawong T., Emasithi A. (2021). Development and validation of the dizziness symptoms questionnaire in Thai-outpatients. Braz. J. Otorhinolaryngol..

[35] Wong F.M.F. (2021). First Data in the Process of Validating a Tool to Evaluate Knowledge, Attitude, and Practice of Healthcare Providers in Oral Care of Institutionalized Elderly Residents: Content Validity, Reliability and Pilot Study. Int. J. Environ. Res. Public Health.

[36] Lynn M.R. (1986). Determination and quantification of content validity. Nurs. Res..

[37] Haynes S.N., Richard D.C.S., Kubany E.S. (1995). Content validity in psychological assessment: A functional approach to concepts and methods. Psychol. Assess..

[38] Sireci S. (1998). The construct of content validity. Soc. Indic. Res..

[39] Sireci S., Faulkner-Bond M. (2014). Validity evidence based on test content. Psicothema.

[40] Shrotryia V.K., Dhanda U. (2019). Content Validity of Assessment Instrument for Employee Engagement. SAGE Open.

[41] Alexandre N.M.C., Coluci M.Z.O. (2011). Validade de conteúdo nos processos de construção e adaptação de instrumentos de medidas. Ciência Saúde Coletiva.

[42] Pilatti L.A., Pedroso B., Gutierrez G.L. (2010). Propriedades Psicométricas de Instrumentos de Avaliação: Um debate necessário. Rev. Bras. Ensino Ciênc. Tecnol..

[43] Sarvimäki A., Stenbock-Hult B. (2016). The meaning of vulnerability to older persons. Nurs. Ethics.

[44] Batchelor-Murphy M., Mcconnell E.S., Amella E.J., Anderson R.A., Bales C.W., Silva S., Barnes A., Beck C., Colon-Emeric C.S. (2017). Experimental Comparison of Efficacy for Three Handfeeding Techniques in Dementia. J. Am. Geriatr. Soc..

